# Alterations in resting-state functional connectivity in patients with Crohn’s disease in remission

**DOI:** 10.1038/s41598-019-43878-0

**Published:** 2019-05-15

**Authors:** Jiancheng Hou, Rosaleena Mohanty, Veena A. Nair, Keith Dodd, Poonam Beniwal-Patel, Sumona Saha, Vivek Prabhakaran

**Affiliations:** 1 Department of Radiology, School of Medicine and Public Health, University of Wisconsin-Madison, Madison, USA; 2 Department of Medicine, Division of Gastroenterology and Hepatology, School of Medicine and Public Health, University of Wisconsin-Madison, Madison, USA; 3 Department of Medicine, Division of Gastroenterology and Hepatology, Medical College of Wisconsin, Milwaukee, USA

**Keywords:** Cognitive neuroscience, Neurological disorders

## Abstract

Previous studies have found neural alterations in regions involved in cognitive and affective functions among Crohn’s disease (CD) patients. The present work recruited 18 CD patients and 18 age-gender matched healthy controls (HC) and specifically compared differences in resting-state functional connectivity (RSFC) within the executive control network (ECN) which has been implicated in cognitive function and default mode network (DMN), which has been implicated in affective function. Additionally, we examined the correlations between RSFC in ECN and verbal fluency (VF) in both groups as well as RSFC in DMN and anxiety level in the CD group. Results showed significantly increased RSFC between the right middle frontal gyrus and right inferior parietal lobule in ECN, as well as increased RSFC between the right precuneus and right posterior cingulate cortex in DMN, among CD patients compared to HC. However, the correlations between ECN/DMN and behavioral scores in each group were not significant, which was possibility due to the limited sample size. These findings suggest that CD patients may experience changes in the connectivity patterns in ECN and DMN. Increased connectivity observed on these networks could be a potential biomarker of a neuropsychiatric manifestation of CD.

## Introduction

Crohn’s disease (CD), one of the main phenotypes of inflammatory bowel disease (IBD), may affect any part of the gastrointestinal tract^1^, secretory gastrointestinal function and the immune system as well as increasing the intestinal permeability^2^. There is also evidence suggesting that the disease may affect the mental state by altering the motor, sensory systems causing psychological stress, precipitating mood disorders, and causing difficulties with concentration and thinking^3^. Compared to age-matched healthy controls, CD patients show brain changes in terms of altered cortical thickness in the bilateral superior and middle frontal gyri, areas (responsible for cognitive control and responding to behaviorally salient events), and the bilateral temporal poles and insular gyri, areas (involved in socio-emotional processing). These morphological changes may be the result of a confluence of factors such as the prolonged exposure to systemic inflammation, pain that may drive changes in the excitatory (e.g., NMDA-ergic) or inhibitory (e.g., GABA-ergic) systems as well as responses to various medications^4^, and could promote CD patients with a heightened sensitivity to their external environment and an inadequate ability to modulate their cognitive and emotional states^4–6^. Additionally, CD patients demonstrate decreased sub-cortical volumes, including the bilateral pallidum and right putamen which are associated with altered emotional and pain perception^4^. These volume changes suggest there is a neural basis to the alterations in CD patients’ cognitive and affective responses. Moreover, a functional neuroimaging study of a language or verbal fluency (VF) task which typically shows a left-lateralized hemispheric activation pattern in young healthy subjects, showed that the duration of CD significantly and positively correlated with verbal fluency task with the activation intensity in key homologous right hemisphere regions suggesting that the disease may lead to accelerated brain changes and compensatory activation patterns in these CD patients^1^.

To our knowledge, limited studies have examined the role of resting-state functional connectivity (RSFC) in CD patients. Liu *et al*. found disrupted local and global topological patterns of functional neural networks (i.e., subcortical, sensorimotor, cognitive control, and default-mode networks) as well as altered local topological patterns associated with clinical characteristics (i.e., anxiety) in CD patients^7^. The connectivity changes in the default mode network during remission period was examined by Thomann *et al*.^8^. However, cognitive neural substrates in CD have largely been unexplored in terms of resting-state functional connectivity. RSFC, the temporal dependency of neuronal activation patterns of anatomically separated brain regions, measures not only the level of co-activation of time-series between brain regions^9^ but also the low-frequency (~0.01–0.1 Hz) spontaneous neuronal activity in the brain^10^ and is believed to reflect neuronal function^11–14^. In the current study, we examined the RSFC patterns in CD patients and compared with age-gender matched healthy control (HC) participants. We focused on the networks involved in cognitive and affective function. Based on results from previous studies, we focused on the executive control network (ECN) and default mode network (DMN), both of which have functions of cognitive control, self-regulation, emotion regulation, and memory suppression^15,16^. The ECN, which includes the regions of the anterior cingulate cortex, anterior prefrontal cortex (aPFC), dorsolateral prefrontal cortex, ventrolateral prefrontal cortex (VLPFC), dorsomedial prefrontal cortex (dmPFC), inferior parietal cortex (IPC), and insula^17,18^, is a less studied network, which constitutes a novel approach to investigate the integrity of brain areas underlying executive function^19^. The DMN, which includes regions of the VLPFC, dmPFC, aPFC, posterior IPC, superior parietal cortex, occipital and temporal cortex^19^ with psychological functions such as attention and cognition^15,20,21^, is a widely studied network that activates in the absence of most external task demands^15,22,23^, but has rarely been examined in CD studies. We hypothesized that the ECN and DMN are impacted by CD and effects might be observable during periods of disease remission. We investigated differences in RSFC between CD patients and their healthy counterparts in these networks. Moreover, we also explored the correlations between ECN connectivity and VF task as well as DMN connectivity and anxiety level.

## Results

### Sample characteristics

The two groups did not significantly differ in education or VF score, as determined by a two-sample t-test and in distribution of gender and handedness, as determined by Fisher’s exact test as specified in Table 1. CD was specially measured by anxiety (BIS and BAS). The distribution of disease location of CD in our cohort was as follows: 55% ileocolonic CD, 25% isolated ileal involvement and 15% isolated colonic disease. Within the CD group, 15% of patients also had perianal disease, and 55% of them had non-structuring and non-penetrating CD.

**Table 1:** Participant Characteristics.

Characteristics	CD	HC	*t*_*(36)*_	*p*
Number	18	18		
Age (years)	35.16 (15.93)	37.28 (18.41)	−0.36	0.72
Education (years)	15.72 (2.78)	15.89 (2.56)	−0.19	0.85
Gender (male/female)	10/8	10/8		1
Handedness (L/R/A)	0/15/3	0/17/1		<0.30
Mean VF score	−0.19 (1.02)	−0.00 (1.19)	−0.51	0.61
Mean BAS score	19.61 (1.83)			
Mean BIS score	25.39 (2.23)			
Mean CES-D Score	32.86 (6.56)	10.64 (6.58)	8.94	0.00
Mean pain score	1.18 (0.82)			
Duration of CD (years)	10.28 (6.44)			
Head motion				
Max (abs (Tx))	0.14 (0.10)	0.18 (0.07)	−1.30	0.20
Max (abs (Ty))	0.25 (0.17)	0.26 (0.21)	−0.18	0.86
Max (abs (Tz))	0.36 (0.21)	0.42 (0.31)	−0.70	0.49
Max (abs (Rx))	0.34 (0.22)	0.67 (0.76)	−1.81	0.08
Max (abs (Ry))	0.23 (0.13)	0.32 (0.37)	−0.98	0.33
Max (abs (Rz))	0.21 (0.15)	0.27 (0.17)	−1.14	0.26
Mean (abs (Tx))	0.07 (0.06)	0.08 (0.04)	−0.65	0.52
Mean (abs (Ty))	0.10 (0.08)	0.10 (0.08)	−0.19	0.85
Mean (abs (Tz))	0.16 (0.12)	0.17 (0.14)	−0.21	0.83
Mean (abs (Rx))	0.16 (0.12)	0.30 (0.37)	−1.60	0.12
Mean (abs (Ry))	0.12 (0.09)	0.16 (0.25)	−0.62	0.54
Mean (abs (Rz))	0.09 (0.07)	0.14 (0.11)	−1.55	0.13
Mean FD Power	0.12 (0.07)	0.14 (0.08)	−0.94	0.35

Note: (1) Standard deviations are shown in parentheses. (2) L: left; R: right; A: ambidextrous. (3) VF: verbal fluency; BAS: behavioral approach system; BIS: behavioral inhibition system; abs: absolute; T: translation; R: rotations.

### RSFC findings

**Figure 1:**
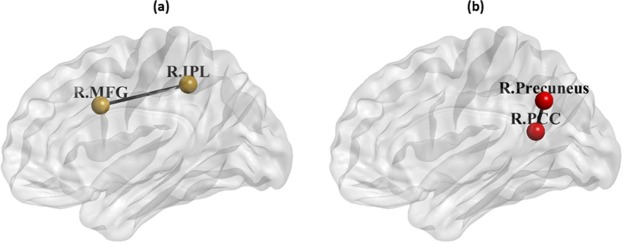
In (**a**) ECN, CD patients had increased RSFC values within the FP subnetwork and in (**b**) DMN, CD patients had increased posterior RSFC values. R: right; MFG: middle frontal gyrus; IPL: inferior parietal lobule; PCC: posterior cingulate cortex.

**Figure 2:**
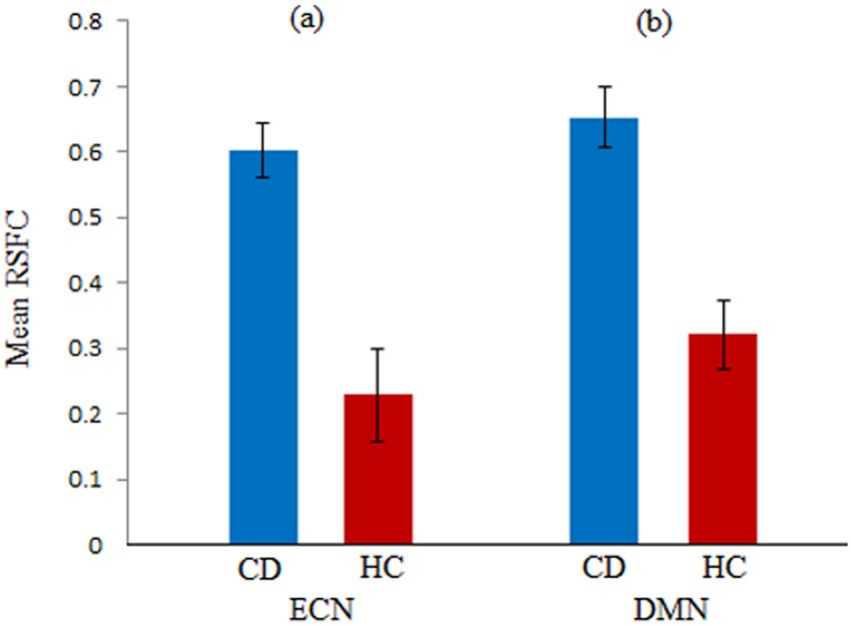
Comparison of mean RSFC in (**a**) ECN (right middle frontal gyrus and right inferior parietal lobule) and (**b**) DMN (right precuneus and right posterior cingulate) for CD and HC groups. Error bars indicate standard errors.

Based on a parametric independent two-sample t-test, significant group differences (FDR corrected *p* < 0.05) between CD and HC were observed within ECN as well as DMN. Within the ECN, RSFC associated with the increased network between the right middle frontal gyrus and right inferior parietal lobule in CD than HC (see Fig. 1a and Table 2). Within the DMN, RSFC associated with the increased network between the right precuneus and right posterior cingulate cortex in CD than HC (see Fig. 1b and Table 2). Thus, in both networks the patients group showed elevated RSFC relative to the control group as exemplified in Fig. 2.

**Table 2:** The RSFC differences between CD patients and HC in ECN and DMN.

Seed 1	Network	Seed 2	Network	Group	Mean (SE)	*t*_*(36)*_	*p*
**ECN**							
Right middle frontal gyrus	ECN	Right inferior parietal lobule	ECN	CD	0.60 (0.04)	4.52	0.046
				HC	0.23 (0.07)		
**DMN**							
Right precuneus	DMN	Right posterior cingulate cortex	DMN	CD	0.65 (0.05)	4.80	0.042
				HC	0.32 (0.05)		

Note: Positive value of T indicate that CD patients have increased RSFC values in comparison to HC. The results are reported as FDR corrected *p* < 0.05. SE: standard error.

### RSFC-behavior relationship

Within both the ECN and DMN that were identified using t-test, the findings from correlation analysis with all behavioral performances did not reach significance, most likely due to the limited sample size.

## Discussion

Limited studies have evaluated RSFC in the CD population^7,8^. Our study provides an addition to this literature and suggests that CD can alter the functional connectivity of the brain. As hypothesized, the ECN and DMN appear to be affected by CD and provides further evidence for gut-brain communication. CD patients exhibited higher levels of RSFC in comparison to HC. Increased RSFC have also been reported in several neuropsychiatric disorders including depression, as well as in people with chronic pain^24^. Our findings are in support of the growing number of studies^4,6,25,26^ that use neuroimaging to examine the neural substrates impacted by CD. MRI, in particular, is a radiation and task-free modality and could complement the standard diagnostic tools for CD such as ileocolonoscopy and gastroduodenoscopy to study the full extent of the impact of CD and potentially better manage its non-gastrointestinal effects^27^.

Our study found alterations in RSFC associated with the right middle frontal gyrus and right inferior parietal lobule in the ECN of CD compared to HC. Specifically, frontal connectivity with the inferior parietal lobule could reflect the functions of top-down control^28^ or spatial attention^29,30^. Although the results of correlations were not significant, the decreased RSFC between frontal connectivity with inferior parietal lobule in HC possibly showed their better performance on VF task. This correspondence is supported by the known involvement of the inferior parietal lobule in attentional control^31^. On the other hand, CD patients exhibited a deviation from this trend with increased frontal RSFC being associated with poorer performance on VF. This deviation may be due to increased levels of inhibitory control by CD patients which may be required to suppress incorrect or irrelevant responses during the VF task^32^.

We also observed increased RSFC involving the right precuneus and right posterior cingulate cortex (both of which belong to a predominantly posterior DMN subsystem^8^) in the CD group compared to HC. Increased RSFC within the posterior DMN was also demonstrated in a recent CD study by Thomann *et al*. who found the association between altered RSFC in DMN and various mental disorders including depression and anxiety disorders^8^. More significant changes in these two regions in CD patients compared to HC as measured by fMRI-based ReHo after undergoing electropuncture treatment has been demonstrated by Bao and colleagues^33^. While our findings from the correlation analysis were not significant, there could be other potential confounders that potentially affect anxiety performances in CD group and it needs further investigation.

This study was limited by a modest sample size. Increasing the sample size would be particularly important to power the correlation analyses for identification of neural correlates linked with behavioral outcomes associated with CD. Our study was based on five-minute long fMRI scans which is considered to be reasonable acquisition time in order to find reliable intrinsic network connectivity^34,35^. However, test-retest reliability studies have also indicated that strength of connectivity can be significantly enhanced with longer acquisition times^36^. This factor, coupled with the sample size may have contributed to limited significant effects in this cohort. Future neuroimaging-based investigations of CD should take this into consideration during the study design. While we controlled for age, gender, handedness and education, there could be other potential confounders that were not included in this analysis. Furthermore, we investigated the ECN and DMN only and thus cannot draw inferences about the effect of CD on other brain networks. Further investigation is needed to assess the impact of cognitive capacity, medications, CD duration, and prior severity of CD on RFSC and to better understand the relationship between RSFC and clinical/behavioral measures as well as RSFC changes over time. While we only focused on the functional connectivity effects occurring in the CD population, a complete characterization would require examination of complementary information from MR such as white matter, grey matter volumes, and potential lesion burden.

This study highlights the utility of resting-state fMRI as a complementary tool to assess the neural manifestations associated with CD. Our results suggest that CD patients in remission exhibit alterations in the connectivity patterns in the ECN and DMN compared to age-gender matched HC. Increased levels of connectivity in CD compared to the HC could serve as potential biomarker for the neuropsychiatric manifestation of CD.

## Methods

### Participants

Nineteen CD patients and nineteen gender- and age-matched healthy controls (HC) were recruited for this study. Because one CD and HC participant each were removed from analysis due to high levels of head motion, we excluded these two participants (so effectively there were eighteen CD patients and eighteen HCs). All participants were recruited on a voluntary basis and provided informed and written consent for participation. Participants were included in the study if they were at least 18 years or older. CD was diagnosed based on endoscopy, histology or radiographic imaging. Participants with CD were in remission based on a Harvey Bradshaw Index^37,38^ score of less than five. Criteria for exclusion were: pregnancy, co-morbid pain disorders unrelated to IBD, scheduled medications for treatment of pain, and contraindications to MRI. A 0–10 rating scale^39–42^ was used to record the intensity of any pain experienced within a week leading up to the study visit in CD patients. The Center for Epidemiologic Studies Depression (CES-D; 20 items) scale^43,44^ was utilized to evaluate symptoms associated with depression. Table 1 shows participants’ basic demographic information.

All methods were carried out in accordance with relevant guidelines and regulations. All experimental protocols were approved by the Institutional Review Board (IRB) of the School of Medicine and Public Health, University of Wisconsin-Madison. Written informed consent was obtained from all participants.

### MRI data acquisition

Five minutes each of resting-state functional MRI and T1 structural MRI data were collected on a 3T MRI scanner (GE750, GE Healthcare, Waukesha, WI). The acquisition parameters were as follows: TR/TE/θ = 2600 ms/22 ms/60°, FOV = 100 × 100 mm, in-plane resolution = 3.5 × 3.5 mm^2^, slice thickness = 3.5mm. Anatomical MRI data were acquired using a T1-weighted, three-dimensional, gradient-echo pulse-sequence (MPRAGE) with TR/TE/θ = 8160 ms/3.18 ms/12°, FOV = 100 × 100 mm, in-plane resolution = 1 × 1 mm^2^, slice thickness = 1 mm. During the scanning, participants laid supine on the scanner bed. Foam pads were used to minimize head motion. Participants were instructed to close their eyes, keep their heads still, and to relax. All participants reported having their eyes closed and being awake during the scan.

### Behavioral data acquisition

To assess the behavioral aspects associated with CD, we acquired measures of verbal fluency (VF) and anxiety to study the neural correlates within ECN and DMN respectively.

#### Verbal Fluency

Controlled Oral Word Association Test (COWAT) was administered to measure verbal fluency (VF)^45^. Participants were required to produce words beginning with the letters “F,” “A,” “S,” in three respective 1-minute trials. The total number of correct responses over the three letters was used to quantify VF. Normalized VF scores achieved on the task were used in subsequent analyses by accounting for age and education levels.

#### Anxiety

Behavioral inhibition system and behavioral approach system (BIS/BAS) scales were administered by questionniare to measure anxiety^46,47^. BIS and BAS scores were calculated for each participant and included 24 items 4-point Likert scale for 20 score-items and 4 fillers). Total scores for BIS (range = 7–28; 7 items) and BAS (range = 13–52; 13 items) were used as outcomes measures of anxiety. While a mean BAS score was computed encompassing domains of reward responsiveness, fun-seeking, and drive, a total BIS score was recorded in response to adverse events.

### Region of interest (ROI) selection

**Figure 3:**
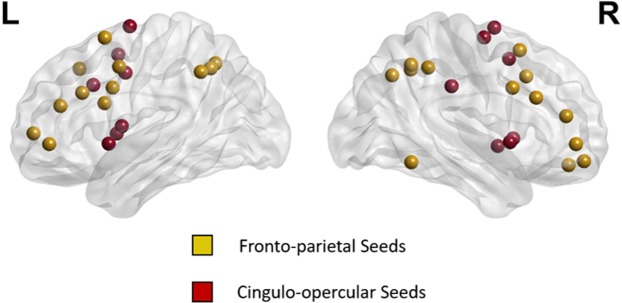
39 regions as seeds in ECN.

**Figure 4:**
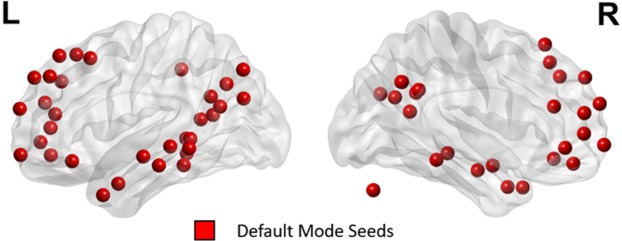
58 regions as seeds in DMN.

Based on a prior study on functional organization of the brain^48^, ROIs in two large-scale brain networks were utilized: (1) 39 ROIs in ECN consisted of 14 ROIs from the cingular-opercular (CO) network and also 25 ROIs from the frontoparietal (FP) network as illustrated in Fig. 3 and Table 3, and (2) 58 ROIs in DMN as shown in Fig. 4 and Table 4. Spherical ROIs, each of radius 5 mm, were created as per the standard MNI coordinates provided by the template.

**Table 3:** The 39 seed regions of the executive control network (ECN).

x	MNI y	z	NETWORK	Anatomical Label
−2.88	2.38	53.21	Cingulo-opercular Task Control	Left Medial Frontal Gyrus & Right BA 6
54.22	−27.83	33.64	Cingulo-opercular Task Control	Right Postcentral Gyrus & Right BA 2
19.33	−7.71	63.88	Cingulo-opercular Task Control	Right Superior Frontal Gyrus & Right BA 6
−16.14	−4.82	70.83	Cingulo-opercular Task Control	Left Superior Frontal Gyrus
−10.48	−2.1	42.02	Cingulo-opercular Task Control	Left Cingulate Gyrus
36.73	0.78	−3.57	Cingulo-opercular Task Control	Right Insula
13.21	−1.36	69.98	Cingulo-opercular Task Control	Right Superior Frontal Gyrus
6.52	7.69	50.58	Cingulo-opercular Task Control	Right Medial Frontal Gyrus
−44.76	0.1	8.83	Cingulo-opercular Task Control	Left Precentral Gyrus & Left BA 44
49.4	8.32	−1.12	Cingulo-opercular Task Control	Right Superior Temporal Gyrus
−34.37	3.29	4.19	Cingulo-opercular Task Control	Left Claustrum
−51.26	8.26	−2.06	Cingulo-opercular Task Control	Left Superior Temporal Gyrus & Left BA 22
−5.33	17.8	34.41	Cingulo-opercular Task Control	Left Cingulate Gyrus
35.83	10.32	1.18	Cingulo-opercular Task Control	Right Insula
−43.93	1.8	45.7	Fronto-parietal Task Control	Left Middle Frontal Gyrus & Left BA 6
47.98	24.56	26.5	Fronto-parietal Task Control	Right Middle Frontal Gyrus
−46.5	10.85	23.04	Fronto-parietal Task Control	Left Inferior Frontal Gyrus
−52.6	−48.83	42.5	Fronto-parietal Task Control	Left Inferior Parietal Lobule
−22.53	10.76	63.73	Fronto-parietal Task Control	Left Superior Frontal Gyrus
58.31	−52.79	−13.61	Fronto-parietal Task Control	Right Inferior Temporal Gyrus & Right BA 20
24.07	44.61	−15.35	Fronto-parietal Task Control	Right Superior Frontal Gyrus & Right BA 11
33.6	54.22	−12.95	Fronto-parietal Task Control	Right Middle Frontal Gyrus & Right BA 11
47.01	9.93	32.66	Fronto-parietal Task Control	Right Middle Frontal Gyrus
−41.06	5.81	32.72	Fronto-parietal Task Control	Left Inferior Frontal Gyrus & Left BA 9
−42.23	38.21	21.35	Fronto-parietal Task Control	Left Middle Frontal Gyrus & Left BA 46
38.37	43.18	15.06	Fronto-parietal Task Control	Right Middle Frontal Gyrus
49.18	−42.41	45.16	Fronto-parietal Task Control	Right Inferior Parietal Lobule
−28.4	−57.93	47.78	Fronto-parietal Task Control	Left Superior Parietal Lobule & Left BA 7
43.93	−52.95	46.95	Fronto-parietal Task Control	Right Inferior Parietal Lobule & Right BA 40
31.83	14.37	55.98	Fronto-parietal Task Control	Right Middle Frontal Gyrus & right BA 6
37.45	−64.7	40.38	Fronto-parietal Task Control	Right Inferior Parietal Lobule
−42.09	−54.98	44.74	Fronto-parietal Task Control	Left Inferior Parietal Lobule & Left BA 40
39.87	18.39	39.72	Fronto-parietal Task Control	Right Middle Frontal Gyrus
−34.16	54.83	4.36	Fronto-parietal Task Control	Left Middle Frontal Gyrus
−41.68	45.16	−2.31	Fronto-parietal Task Control	Left Middle Frontal Gyrus & Left BA 10
33.38	−53.12	44.02	Fronto-parietal Task Control	Right Inferior Parietal Lobule
43.25	49.25	−2.31	Fronto-parietal Task Control	Right Middle Frontal Gyrus
−42.1	24.68	29.53	Fronto-parietal Task Control	Left Middle Frontal Gyrus & Left BA 9
−2.98	26.41	44.42	Fronto-parietal Task Control	Left Medial Frontal Gyrus & Left BA 8

**Table 4:** The 58 seed regions of the default mode network (DMN).

MNI X	MNI Y	MNI Z	NETWORK	Anatomical Label
−40.5	−75.27	25.8	Default mode	Left Superior Occipital Gyrus
5.55	66.69	−3.55	Default mode	Right Medial Frontal Gyrus
8.36	47.59	−15.18	Default mode	Right Medial Frontal Gyrus
−12.6	−39.64	0.93	Default mode	Left Parahippocampal Gyrus & Left BA 30
−17.65	63.19	−9.17	Default mode	Left Superior Frontal Gyrus
−45.79	−60.69	20.85	Default mode	Left Superior Temporal Gyrus & Left BA 39
43.43	−72.21	28	Default mode	Right Middle Temporal Gyrus & Right BA 39
−43.58	11.99	−34.15	Default mode	Left Middle Temporal Gyrus
45.64	16.2	−30.02	Default mode	Right Superior Temporal Gyrus
−68.47	−22.66	−15.74	Default mode	Left Middle Temporal Gyrus
−44.45	−64.64	34.78	Default mode	Left Angular Gyrus & Left BA 39
−39.05	−74.95	43.72	Default mode	Left Precuneus
−6.84	−54.9	27.05	Default mode	Left Cingulate Gyrus & Left BA 31
5.91	−58.82	35.45	Default mode	Right Precuneus
−11.29	−56.2	15.6	Default mode	Left Posterior Cingulate
−2.94	−48.79	12.87	Default mode	Left Posterior Cingulate & Left BA 29
7.94	−48.37	30.57	Default mode	Right Precuneus & Right BA 31
15.12	−63.09	25.98	Default mode	Right Precuneus & Right BA 31
−2.2	−36.68	43.85	Default mode	Left Precuneus & Left BA 7
10.77	−53.83	17.09	Default mode	Right Posterior Cingulate
52.04	−59.37	35.52	Default mode	Right Angular Gyrus
23.33	33.07	47.68	Default mode	Right Superior Frontal Gyrus & Right BA 8
−10.09	39.09	52.29	Default mode	Left Superior Frontal Gyrus & Left BA 8
−16.4	28.52	53.05	Default mode	Left Superior Frontal Gyrus & Left BA 6
−35.36	19.86	50.8	Default mode	Left Superior Frontal Gyrus
22.11	39.21	38.9	Default mode	Right Superior Frontal Gyrus & Right BA 8
12.73	54.87	38.19	Default mode	Right Superior Frontal Gyrus
−10.33	54.63	38.71	Default mode	Left Superior Frontal Gyrus
−19.78	45.07	39.48	Default mode	Left Superior Frontal Gyrus
5.94	54.42	16.18	Default mode	Right Medial Frontal Gyrus & Right BA 9
6.11	63.98	21.96	Default mode	Right Superior Frontal Gyrus
−7.04	50.82	−1.29	Default mode	Left Anterior Cingulate & Left BA 10
8.8	54.23	3.45	Default mode	Right Medial Frontal Gyrus & Right BA 10
−3.06	44.41	−9.46	Default mode	Left Medial Frontal Gyrus & Left BA 10
7.51	42.49	−5.35	Default mode	Right Anterior Cingulate
−11.06	44.62	7.61	Default mode	Left Anterior Cingulate
−2.06	37.85	36.34	Default mode	Left Medial Frontal Gyrus & Left BA 6
−2.5	41.7	16.05	Default mode	Left Medial Frontal Gyrus
−20.16	63.65	19.39	Default mode	Left Superior Frontal Gyrus & Left BA 10
−7.55	48.08	23.18	Default mode	Left Medial Frontal Gyrus & Left BA 9
64.64	−11.8	−19.3	Default mode	Right Inferior Temporal Gyrus
−55.72	−12.96	−10.24	Default mode	Left Middle Temporal Gyrus
−57.75	−29.7	−3.94	Default mode	Left Middle Temporal Gyrus
64.8	−30.55	−8.7	Default mode	Right Middle Temporal Gyrus
−68.3	−41.41	−5.14	Default mode	Left Middle Temporal Gyrus
13.08	29.99	58.65	Default mode	Right Superior Frontal Gyrus
12.25	35.63	20.3	Default mode	Right Anterior Cingulate & Right BA 32
52.16	−2.43	−16.4	Default mode	Right Middle Temporal Gyrus
−26.44	−39.95	−8.26	Default mode	Left Parahippocampal Gyrus & Left BA 36
26.94	−37.34	−12.76	Default mode	Right Parahippocampal Gyrus
−33.93	−38.06	−15.6	Default mode	Left Fusiform Gyrus & Left BA 20
28.46	−76.56	−31.64	Default mode	Right Pyramis
51.9	6.81	−29.61	Default mode	Right Middle Temporal Gyrus & Right BA 21
−52.89	2.55	−27.06	Default mode	Left Middle Temporal Gyrus & Left BA 21
46.68	−50.08	28.76	Default mode	Right Supramarginal Gyrus
−49.3	−42.15	0.83	Default mode	Left Middle Temporal Gyrus
−46.17	31.26	−13.03	Default mode	Left Inferior Frontal Gyrus & Left BA 47
49.26	35.47	−12.2	Default mode	Right Inferior Frontal Gyrus

### Data preprocessing

The RSFC analysis was performed using the Data Processing Assistant for Resting-state fMRI Basic Edition toolbox (DPARSF V4.3), which is part of the Data Processing and Analysis of Brain Imaging (DPABI) toolbox version 3.1 (http://rfmri.org/dpabi)^49,50^. DPARSF is a convenient plug-in software based on Statistical Parametric Mapping (SPM) and Resting-State fMRI Data Analysis Toolkit (REST) (http://www.restfmri.net) integrated in Matlab^50^. The Digital Imaging and Communications in Medicine (DICOM) files were first arranged and the parameters (such as time points, TR, slice number, voxel size *et al*.) were then set. DPARSF then produced the preprocessed data (with slice timing, realignment, normalization, and smoothing). The first five volumes were discarded to allow the magnetization to approach a dynamic equilibrium, and for the participants to get used to the scanner noise. Data pre-processing, including slice timing, realignment, normalization, smoothing, regressing out head motion parameters (scrubbing within Friston 24-parameter model regression; bad time points were identified using a threshold of frame-wise displacement (Power FD) > 0.2 mm as well as 1 volume before and 2 volumes after at the individual-subject level as well as accounting for head motion at the group-level (i.e., covariate analysis))^49,51,52^, and spatial normalization to the Montreal Neurological Institute (MNI) template (resampling voxel size of 3.5 × 3.5 × 3.5 mm), were conducted using SPM8 and DPARSF version 4.3^50,53^. A spatial Gaussian filter of 4 mm FWHM (full-width at half maximum) was used for smoothing. We calculated the temporal correlations as spontaneous neuronal connectivity to quantify RSFC and generated a 39 × 39 symmetric correlation matrix for ECN and a 58 × 58 symmetric correlation matrix for DMN per participant for CD and HC groups. From these matrices, a total of 741 and 1653 unique pairwise functional connections were extracted from the ECN and DMN, respectively, for each participant.

### Statistical analysis

Group differences between RSFC of CD and HC groups were examined using independent two-sample t-test in IBM SPSS version 23, with head motion used as a covariate. All results were corrected for multiple comparisons by estimating the false discovery rate (FDR)^54^ based on the procedure in Matlab R2016b (The MathWorks, Inc., Natick, Massachusetts, United States). Specific functional connections with corrected *p-value* < 0.05 were deemed to be significantly different between CD and HC groups in each network. Additionally, exploratory analyses were performed by correlating the identified RSFC connections from t-tests: (1) in ECN with performance on VF task in CD and HC groups, and (2) in DMN with anxiety measures in CD group only. Significant brain connections identified by the t-test were used to perform correlation analyses based on Pearson’s correlation using IBM SPSS version 23. The group differences were visualized with the BrainNet Viewer Toolbox^55^.
